# Detecting childhood trauma in MDD patients through automated speech and language analysis

**DOI:** 10.1192/j.eurpsy.2025.1188

**Published:** 2025-08-26

**Authors:** A. König, E. Ettore, H. Lindsay, J. Tröger, M. Benoit, P. Robert

**Affiliations:** 1 ki:elements, Saarbrücken, Germany; 2 Université Côte d’Azur, Centre Hospitalier et Universitaire, Clinique Gériatrique du Cerveau et du Mouvement, Centre Mémoire de Ressources et de Recherche; 3 Université Côte d’Azur, Centre Hospitalier et Universitaire, Département de Psychiatrie, Nice, France; 4 CobTek lab, Université Côte d’Azur, Nice, France

## Abstract

**Introduction:**

Speech patterns offer valuable insights into cognitive and emotional states, particularly in mental health conditions such as Major Depressive Disorder (MDD). Traditional assessments often fail to capture its full severity, prompting the need for objective, non-invasive tools.

**Objectives:**

The objective of this study is to explore the potential of automatic speech analysis as a new method for distinguishing between varying severities of Major Depressive Disorder (MDD), and to assess how childhood trauma may further influence speech characteristics in individuals with depression.

**Methods:**

Participants were recruited from the psychiatric clinic at the University Hospital in Nice, France. The cohort consisted of 27 patients diagnosed with MDD, divided into mild-to-moderate and severe depression groups based on Beck Depression Inventory (BDI) and Montgomery–Åsberg Depression Rating Scale (MADRS) scores. Speech recordings from semi-structured (V0) and free (V1) clinical interviews were analyzed using automatic speech recognition and feature extraction. Linguistic, prosodic, and spectral features were examined. Additionally, childhood trauma was assessed using the Childhood Trauma Questionnaire (CTQ), and associations with speech characteristics were explored.

**Results:**

In the severe depression group, longer pause durations and lower word frequency were observed in V0 interviews. Word frequency and proper noun usage were significantly different between groups, but the small differences in means made interpretation difficult. Free speech analysis (V1) showed that more severe depression correlated with fewer repetitions and reduced semantic richness. In BDI-based analysis, severe depression was associated with lower F2 frequency and bandwidth, alongside lower Harmonics-to-Noise Ratio (HNR), which persisted in both V0 and V1. Prosodic parameters revealed less speech duration and articulatory effort in severe cases. Analysis of childhood trauma showed that traumatic load correlated with longer speaking time and greater discourse complexity, in contrast to depression severity, which was associated with shorter speech and fewer repetitions.

**Conclusions:**

Speech parameters, particularly pause duration and word frequency, demonstrate potential for distinguishing depression severity. Childhood trauma influences linguistic complexity, suggesting different underlying mechanisms between trauma and depression. Further studies are needed to validate these findings and explore the clinical applicability of speech analysis in psychiatric assessments.

**Disclosure of Interest:**

A. König Employee of: Employee at the company ki:elements, E. Ettore: None Declared, H. Lindsay: None Declared, J. Tröger: None Declared, M. Benoit: None Declared, P. Robert : None Declared

